# Carbohydrate-to-fat ratio is positively associated with comprehensive frailty across protein-intake levels in older adults

**DOI:** 10.1007/s00394-026-04099-0

**Published:** 2026-10-07

**Authors:** Hinako Nanri, Tsukasa Yoshida, Eiichi Yoshimura, Jun Kunisawa, Hiroyuki Fujita, Misaka Kimura, Yosuke Yamada

**Affiliations:** 1 National Institutes of Biomedical Innovation, Health and Nutrition, 3-17 Senrioka-shinmachi, Settsu-shi, Osaka, 566-0002 Japan; 2 Institute for Active Health, Kyoto University of Advanced Science, Kyoto, Japan; 3 Graduate School of Medicine, Tohoku University, Miyagi, Japan

**Keywords:** Comprehensive frailty, Carbohydrate-to-fat ratio, Protein intake, Older adults, Kihon checklist

## Abstract

**Purpose:**

Diet influences frailty, and protein intake has been a prime focus of prevention. However, frailty remains prevalent, even among older adults with relatively high protein intake, suggesting the influence of remaining energy distribution between carbohydrates and fats. We investigated the association between the carbohydrate-to-fat energy ratio (CFr) and comprehensive frailty in older Japanese adults and whether this association differed by protein intake.

**Methods:**

Cross-sectional baseline data from 5679 community-dwelling adults aged ≥ 65 years in the Kyoto–Kameoka Study were analyzed. Diet was assessed using a validated 46-item food-frequency questionnaire. Percent energy from carbohydrates, fats, and protein was calculated. CFr was defined as the ratio of percent energy from carbohydrates to that from fat. Frailty was defined as a Kihon Checklist score ≥ 7. Multivariable logistic regression estimated odds ratios (ORs) and 95% confidence intervals (CIs) for frailty across CFr quartiles, adjusting for sociodemographic, lifestyle, and health-related factors. Restricted cubic splines assessed dose–response associations overall and within sex-specific tertiles of protein intake.

**Results:**

Among the 5679 participants, 1705 (30.0%) had comprehensive frailty. Compared with the lowest CFr quartile, the adjusted ORs were 1.12 (95% CI, 0.92–1.36) for Q2, 1.44 (1.18–1.76) for Q3, and 1.92 (1.55–2.38) for Q4 (*P* for trend < 0.001). Positive associations were also observed across sex-specific protein-intake tertiles, with no evidence of effect modification by protein intake.

**Conclusion:**

A higher CFr is associated with a higher prevalence of comprehensive frailty in older Japanese adults, with broadly similar associations across protein-intake levels.

**Supplementary Information:**

The online version contains supplementary material available at https://doi.org/10.1007/s00394-026-04099-0.

## Introduction

Amid global population aging, frailty is a multidimensional geriatric syndrome characterized by diminished physiological reserves and impaired recovery homeostasis after stress [1]. Frailty encompasses physical, cognitive, and social domains that interact to create complex health vulnerabilities. Frailty is associated with greater needs for long-term care, increased risk of hospital admission, and higher mortality [2]. As frailty can be slowed or prevented when identified early, clarifying modifiable determinants is a public-health priority; among these, diet is a key, tractable risk factor [3]. Comprehensive frailty, which captures not only physical vulnerability but also cognitive and social deficits, may better reflect the real-world risk of functional decline and loss of independence than physical frailty alone.

Although protein intake has been a prime focus of frailty prevention, a “protein-focused” strategy alone may be insufficient to meaningfully lower the risk of frailty. Adequate nutritional intake, particularly sufficient protein, helps preserve muscle mass and strength and is consistently associated with a lower prevalence of physical and comprehensive frailty [4–8]. However, a cross-sectional study of 5679 Japanese adults aged ≥ 65 years indicated that the prevalence of comprehensive frailty defined by the Kihon Checklist remained approximately 25–30%, even in the highest quartile of protein intake [7, 8], indicating that a substantial frailty burden persists despite relatively high protein intake. This residual burden suggests that factors beyond protein intake may also be relevant to comprehensive frailty. Additionally, the feasible contribution of protein to total metabolizable energy is relatively narrow, typically being approximately 10–20% of energy in human diets and well below a putative upper tolerance of approximately 35% [9, 10]. In contrast, total digestible carbohydrate and total fat together account for approximately 80–85% of energy intake and span a much wider range (approximately 10–75% of energy) [11–14]. These features suggest that, once adequate protein is secured, the partitioning of the remaining energy between carbohydrates and fats may represent an additional dimension of dietary patterns relevant to frailty.

Findings regarding the association between total carbohydrate or fat intake and frailty have been inconsistent [15, 16]. Cross-sectional studies using low-carbohydrate dietary scores suggest that dietary patterns characterized by a lower carbohydrate contribution and a higher fat contribution may be associated with lower odds of frailty [17, 18]. However, carbohydrate and fat intake are interdependent components of dietary energy allocation and examining either nutrient in isolation may not adequately represent their relative balance. The carbohydrate-to-fat energy ratio (CFr), calculated as carbohydrate-derived energy divided by fat-derived energy, provides a simple directional indicator of this balance, with a higher value reflecting a greater contribution of carbohydrates relative to fats. Previous studies have reported that higher carbohydrate intake or carbohydrate-dominant dietary patterns are associated with a higher prevalence or severity of frailty in older adults. [17, 18] In addition, a higher dietary carbohydrate-to-fat ratio is associated with poorer cognitive performance in older adults at risk of dementia [19], suggesting that the relative allocation of energy between carbohydrates and fats may be relevant to multiple dimensions of health in later stages of life. Nevertheless, the association between CFr and multidimensional frailty has not been well characterized.

The primary aim and novelty of this study were to evaluate the association of an energy-based CFr with comprehensive frailty assessed using the Kihon Checklist. We also examined whether this association was consistent across sex-specific protein-intake tertiles among community-dwelling older Japanese adults. We hypothesized that a higher CFr would be associated with higher odds of comprehensive frailty and that this association would be consistent across protein-intake strata.

## Methods

### Study participants

The Kyoto–Kameoka study was a prospective cohort study that focused on incidents of falls and accidents and the reduction of long-term care needs among individuals aged ≥ 65 years in Kameoka City, Kyoto Prefecture, Japan [20]. In July 2011, a comprehensive survey, the Needs in the Sphere of Daily Life Survey (baseline survey), which included the 25-item Kihon Checklist (KCL), was conducted. Valid responses were received from 13,294 participants (response rate, 72.9%). Six months later, in February 2012, an additional Health and Nutritional Survey that included a dietary survey was conducted and yielded valid responses from 8319 participants (valid response rate, 69.4%). Both surveys were administered via postal questionnaires, and informed consent was obtained from those who returned the completed forms. The study was approved by the Ethics Committees of Kyoto University of Advanced Science (No. 24M20) and National Institute of Biomedical Innovation, Health and Nutrition (2025–035).

Of the 8319 participants, 2640 were excluded sequentially: 474 because of missing or invalid height or weight data required to calculate BMI, 205 because of implausible total energy intake (< 500 or > 4000 kcal/day for men; < 500 or > 3500 kcal/day for women), and 1961 because of incomplete responses to one or more of the 25 Kihon Checklist items. The exclusion criteria were applied hierarchically in the given order, and each participant was counted only once at the first applicable exclusion step. Missing values for categorical covariates were retained as explicit “Missing” categories and did not result in additional exclusions. The final analytic sample comprised 5679 participants (2722 men and 2957 women). The same analytic sample was used for all primary analyses, including models fitted in the overall population and analyses conducted within sex-specific tertiles of protein intake. The participant-selection process is shown in Supplementary Figure S1.

### Dietary assessment

Dietary intake was evaluated using a validated self-administered food frequency questionnaire [21–25]. Participants were asked to report the frequency of their consumption of 46 food and beverage items (including green tea and coffee) over the previous year to estimate their usual intake levels. Total energy and nutrient intakes were calculated using a program developed by the Department of Public Health, Nagoya City University School of Medicine [21–24], based on the Standard Tables of Food Composition in Japan (fifth revised edition) [26]. Energy derived from protein, fats, and carbohydrates was calculated directly from the estimated intake of each macronutrient using energy conversion factors of 4, 9, and 4 kcal/g, respectively. The percentage of total energy derived from each macronutrient was calculated by dividing the corresponding macronutrient-derived energy by total energy intake and multiplying by 100. CFr was calculated directly as carbohydrate-derived energy divided by fat-derived energy, consistent with the method used in a recent population-based study [27]. This definition is algebraically represented as eCarb/eFat. Body-weight-normalized protein intake was calculated by dividing estimated protein intake (g/day) by body weight (kg) and was expressed as g/kg body weight/day.

### Definition of comprehensive frailty

The KCL is a validated tool widely used to screen for frailty among community-dwelling older adults [28, 29]. It comprises 25 items grouped into seven subdomains: (i) questions 1–5 assess instrumental activities of daily living; (ii) questions 6–10 assess physical function; (iii) questions 11–12 assess nutritional status; (iv) questions 13–15 assess oral function; (v) questions 16–17 assess housebound and socialization status; (vi) questions 18–20 assess cognitive function; and (vii) questions 21–25 assess depressive mood. Each item was scored as 0 or 1 as per the established scoring rules, with 1 indicating a frailty-related response. The 25 individual-item scores were summed to obtain a total score ranging from 0 to 25, with higher scores indicating greater multidimensional impairment. Comprehensive frailty was defined as a total KCL score of ≥ 7 [8, 30, 31]. The total score was calculated only when valid responses were available for all 25 items. If one or more items were missing or invalid, the total KCL score was treated as missing; no imputation, prorating, or substitution was performed.

### Covariates

Additional data were collected via a self-administered questionnaire, which included questions on height, weight, family structure, education, economic difficulty, history of diseases (e.g., cardiovascular disease and stroke), alcohol consumption, smoking status, and self-reported health. Economic difficulty was assessed using a self-reported question on perceived financial status and dichotomized as difficult (very difficult or somewhat difficult) or not difficult (somewhat comfortable or comfortable). Alcohol consumption was categorized as daily, sometimes, seldom, or never. Smoking status was classified as daily, sometimes, former, and never. Self-reported health was rated as very good, good, fair, or poor, then grouped into very good/good or fair/poor. Participants’ ages were calculated based on their date of birth recorded in the city office’s resident register. Body mass index (BMI) was calculated by dividing self-reported weight (kg) by the square of self-reported height (m). The population density was calculated as the number of residents per unit area (km^2^).

### Statistical analysis

First, the association between CFr and comprehensive frailty was evaluated in the overall analytic sample. CFr was categorized into quartiles using cut-points corresponding to the 25th, 50th, and 75th percentiles of its distribution in the overall analytic sample (1.69, 2.26, and 3.02, respectively). Then, participants were stratified according to sex-specific tertiles of protein intake, expressed as a percentage of total energy intake. The tertile cut-points were 10.9% and 12.4% for men and 12.1% and 14.0% for women. The association between CFr and comprehensive frailty was re-estimated within each protein-intake tertile to assess whether the association was consistent across protein-intake levels.

Baseline characteristics were summarized overall and according to protein-intake tertile. Categorical variables were presented as numbers and percentages, and continuous variables as means with standard deviations or medians with interquartile ranges, as appropriate. Differences across protein-intake tertiles were examined using Pearson’s chi-squared test for categorical variables and the Kruskal–Wallis rank-sum test for continuous variables.

Multivariable logistic regression was used to estimate odds ratios (ORs) and 95% confidence intervals (CIs) for prevalent comprehensive frailty across CFr quartiles, with the lowest quartile (Q1) as the reference. Models were adjusted for sex (male or female), age (years, continuous), BMI (kg/m^2^, continuous), total energy intake (kcal/day, continuous), alcohol consumption status (every day, sometimes, seldom, or never), smoking status (current daily, current occasional, former, or never), history of disease (hypertension, diabetes, dyslipidemia, cancer, cardiovascular disease, stroke, liver disease, or chronic renal failure; yes or no), family structure (living alone, living with others, or other), educational attainment (≤ 9, 10–12, or ≥ 13 years), economic difficulty (yes or no), population density (≥ 1000 or <1000 persons/km^2^), and self-rated health (good or poor). Missing values for categorical covariates were retained as explicit “Missing” categories.

Within each sex-specific protein-intake tertile, the association between CFr and comprehensive frailty was re-estimated using the same covariate set as in the main model. Linear trends across CFr quartiles were assessed in the overall sample and within each protein-intake tertile by assigning each participant the median CFr value of their quartile and entering this value as a continuous term in the model.

Restricted cubic spline logistic regression models were fitted to characterize the dose–response association between CFr and comprehensive frailty. Three knots were placed at the 10th, 50th, and 90th percentiles of the CFr distribution in the overall analytic sample and were retained in the protein-intake-stratified analyses. The reference was anchored at the median CFr of Q1. Predicted adjusted ORs with 95% CIs were plotted across the observed range. Overall associations and departures from linearity were evaluated using Wald χ^2^ tests. Effect modification by protein-intake tertile was evaluated using a likelihood-ratio test comparing models with and without interaction terms between protein-intake tertile and the CFr spline terms.

As a sensitivity analysis, participants were categorized according to body-weight-normalized protein intake using a cutoff of 1.0 g/kg body weight/day (< 1.0 or ≥ 1.0 g/kg body weight/day). Restricted cubic spline analyses were repeated within these categories using the same knot locations, reference value, and covariate set as in the primary analyses. As an additional sensitivity analysis, restricted cubic spline analysis was repeated among non-current drinkers to minimize the potential influence of alcohol-derived energy on macronutrient energy composition. Alcohol consumption status was not included as a covariate in this analysis.

In exploratory domain-specific analyses, impairment in each KCL domain was defined using established cutoffs: instrumental activities of daily living (questions 1–5, score ≥ 3), physical function (questions 6–10, score ≥ 3), nutritional status (questions 11–12, score = 2), oral function (questions 13–15, score ≥ 2), housebound/socialization status (question 16, response of “No”), cognitive function (questions 18–20, score ≥ 1), and depressive mood (questions 21–25, score ≥ 2). Multivariable logistic regression models were used to estimate ORs and 95% CIs for each domain-specific impairment per 1-unit increase in CFr, using the same covariate set as in the primary analysis.

To assess potential conceptual overlap between dietary intake and the nutritional-status and oral-function components of the KCL, an exploratory sensitivity analysis was conducted using a modified 20-item KCL score that omitted questions 11–15. Participants were not excluded on the basis of their responses to these items; rather, only the five items were omitted from the score calculation. A modified score of ≥7 was used as an exploratory cutoff. Quartile-based logistic regression and restricted cubic spline analyses were repeated using this modified outcome and the same covariate set as in the primary analysis. Because the modified outcome and cutoff have not been formally validated, the results were interpreted cautiously.

All tests were two-sided, with a significance level of 0.05. Statistical analyses were performed using R version 4.1.2 (R Foundation for Statistical Computing, Vienna, Austria).

## Results

Among the 5679 participants, the mean KCL score was 5.05 (SD, 4.19), the median was 4 (IQR, 2–7), and the scores ranged from 0 to 23; 1705 participants (30.0%) had a KCL score ≥7. The prevalence of comprehensive frailty increased from 26.1% in CFr Q1 to 36.1% in Q4 and was 33.0%, 27.7%, and 29.4% across protein-intake tertiles T1, T2, and T3, respectively (Supplementary Figure S2 and Supplementary Table S1).

Table 1 summarizes the baseline characteristics of the 5679 participants overall and according to sex-specific tertiles of protein intake. Overall, 2957 participants (52.1%) were women, the mean age was 72.3 years (SD, 5.9 years), the mean body mass index was 22.7 kg/m^2^ (SD, 3.3 kg/m^2^), and the mean total energy intake was 1757 kcal/day (SD, 434 kcal/day). Baseline characteristics were generally comparable across protein-intake tertiles, although total energy intake was lower in the highest than in the lowest tertile.

**Table 1:** Baseline characteristics overall and sex-specific tertiles of protein (% energy)

Baseline characteristics	Overall	Protein intake % energy		
		Tertile 1 (Low) n = 1,894	Tertile 2 (Middle) n = 1,892	Tertile 3 (High) n = 1,893
Women, n (%)	2957 (52.1)^a^	986 (52.1)	986 (52.1)	985 (52.1)
Age, years	72.7 (5.9)	72.6 (6.0)	72.3 (5.8)	73.2 (5.9)
Height, cm	157.7 (8.8)	157.6 (8.9)	157.7 (8.6)	157.7 (8.7)
Weight, kg	56.6 (10.6)	56.4 (10.1)	56.6 (10.3)	56.9 (11.3)
Body mass index, kg/m^2^	22.7 (3.3)	22.6 (3.1)	22.7 (3.2)	22.8 (3.7)
Living alone, n (%)	616 (11.4)	227 (12.6)	178 (9.9)	211 (11.8)
Education attainment ≥ 13 years, n (%)	1325 (25.0)	405 (23.1)	429 (24.0)	491 (27.9)
Economic difficulty, n (%)	3576 (64.6)	1230 (66.3)	1195 (64.9)	1,151 (62.6)
Population density ≥ 1000 people/km^2^, n (%)	2642 (46.5)	812 (42.9)	918 (48.5)	912 (48.2)
History of disease, n (%)	3526 (62.1)	1172 (61.9)	1179 (62.3)	1175 (62.1)
Current drinker, n (%)	2442 (43.2)	872 (46.2)	798 (42.4)	772 (41.0)
Current smoker, n (%)	606 (10.7)	218 (11.6)	195 (10.4)	193 (10.3)
Poor self-reported health, n (%)	919 (16.3)	297 (15.8)	307 (16.4)	315 (16.8)
Total energy intake, kcal/day	1757 (434)	1831 (467)	1759 (375)	1681 (442)

^a^Values are n (%) for categorical variables and mean (SD) for continuous variables

**Fig. 1:**
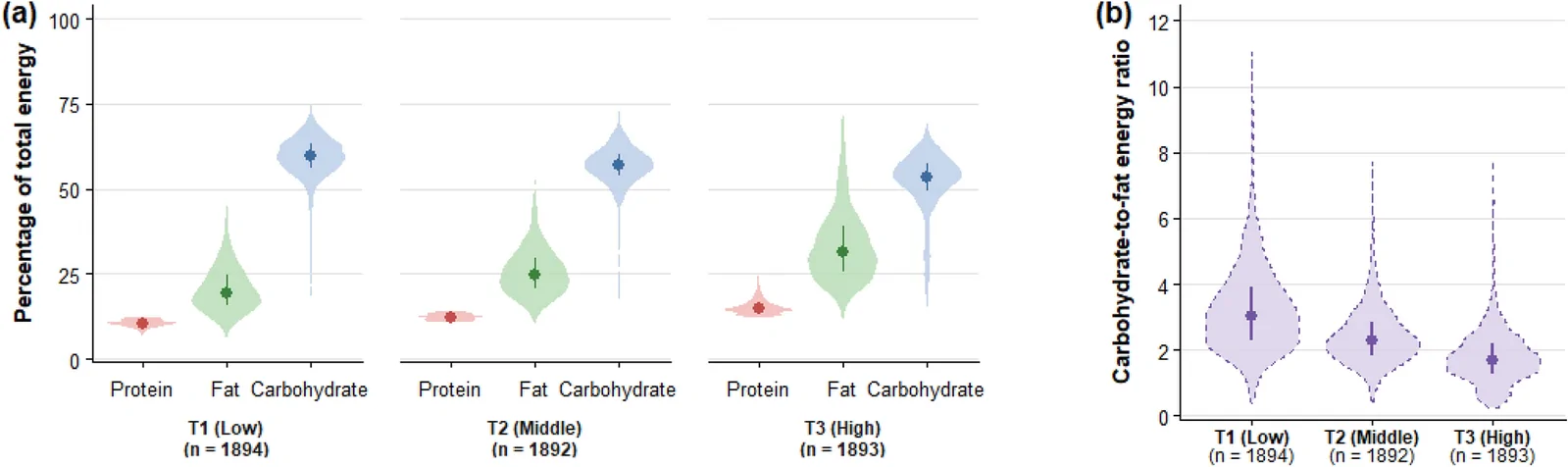
Distributions of macronutrient energy percentages and the carbohydrate-to-fat energy ratio according to sex-specific protein-intake tertiles. Panel A shows the distributions of the percentages of total energy intake derived from protein, fat, and carbohydrate, whereas Panel B shows the distribution of the carbohydrate-to-fat energy ratio (CFr). Protein-intake tertiles were defined separately for men and women. Violin plots represent the distribution of each variable; points and vertical lines indicate the median and interquartile range, respectively

Figure 1 shows violin plots of the percentages of total energy derived from protein, fats, and carbohydrates, together with CFr, according to sex-specific protein-intake tertile. As protein intake increased from T1 to T3, the median carbohydrate share decreased and median fat share increased, with substantial but overlapping distributions across tertiles. Consequently, CFr declined stepwise from the low- to high-protein groups.

Table 2 shows that higher CFr quartiles were associated with higher odds of comprehensive frailty in the overall sample. Compared with Q1, the adjusted OR was 1.92 (95% CI, 1.55–2.38) for Q4, with a significant linear trend (*P* for trend <0.001). Similar positive trends were observed across all sex-specific protein-intake tertiles (*P* for trend < 0.001, 0.003, and 0.004 for T1, T2, and T3, respectively).

**Table 2:** Multivariable-adjusted odds ratios (ORs) and 95% confidence intervals (CIs) for comprehensive frailty assessed using the Kihon Checklist according to quartiles (Q) of carbohydrate-to-fat ratio (CFr), overall and stratified by sex-specific tertiles (T) of protein intake

	Q1	Q2	Q3	Q4	*P*^a^
	Reference	OR (95% CI)	OR (95% CI)	OR (95% CI)	
All, n	1420	1420	1419	1420	
Comprehensive frailty, n (%)	370 (26.1) 53 (37.3)	380 (26.8)	443 (31.2)	512 (36.1)	
Model	1.00^b^	1.12 (0.92, 1.36)	1.44 (1.18, 1.76)	1.92 (1.55, 2.38)	<0.001
Protein intake category					
T1 (Low), n	135	316	489	954	
Comprehensive frailty, n (%)	42 (31.1) 53 (37.3)	77 (24.4)	153 (31.3)	353 (37.0)	
Model	1.00^b^	0.89 (0.51, 1.53)	1.30 (0.78, 2.16)	1.73 (1.04, 2.88)	<0.001
T2 (Middle), n	339	593	589	371	
Comprehensive frailty, n (%)	84 (24.8) 53 (37.3)	145 (24.5)	174 (29.5)	121 (32.6)	
Model	1.00^b^	0.97 (0.68, 1.38)	1.48 (1.02, 2.16)	1.77 (1.13, 2.77)	0.003
T3 (High), n	946	511	341	95	
Comprehensive frailty, n (%)	244 (25.8) 53 (37.3)	158 (30.9)	116 (34.0)	38 (40.0)	
Model	1.00^b^	1.40 (1.04, 1.88)	1.52 (1.07, 2.15)	1.94 (1.12, 3.37)	0.004

^a^*P* for trend across CFr quartiles was calculated by assigning participants the median CFr value of their quartile and entering this value as a continuous term in the multivariable logistic regression model

^b^Odds ratios and 95% confidence intervals were estimated using multivariable logistic regression models adjusted for sex (male or female), age (years, continuous), body mass index (kg/m^2^, continuous), total energy intake (kcal/day, continuous), alcohol consumption status (every day, sometimes, seldom, or never), smoking status (current daily, current occasional, former, or never), history of one or more of the following diseases (hypertension, diabetes, dyslipidemia, cancer, cardiovascular disease, stroke, liver disease, or chronic renal failure; yes or no), family structure (living alone, living with others, or other), educational attainment (≤ 9, 10–12, or ≥ 13 years), economic difficulty (yes or no), population density (≥ 1000 or < 1000 persons/km^2^), and self-rated health (good or poor). Missing values for categorical covariates were included as separate “Missing” categories

CFr quartiles were defined using cut-points corresponding to the 25th, 50th, and 75th percentiles of the CFr distribution in the overall analytic sample: 1.69, 2.26, and 3.02, respectively. Protein-intake tertiles were defined separately by sex using cut-points of 10.9% and 12.4% energy for men and 12.1% and 14.0% energy for women. CFr, carbohydrate-to-fat energy ratio; CI, confidence interval; OR, odds ratio

**Fig. 2:**
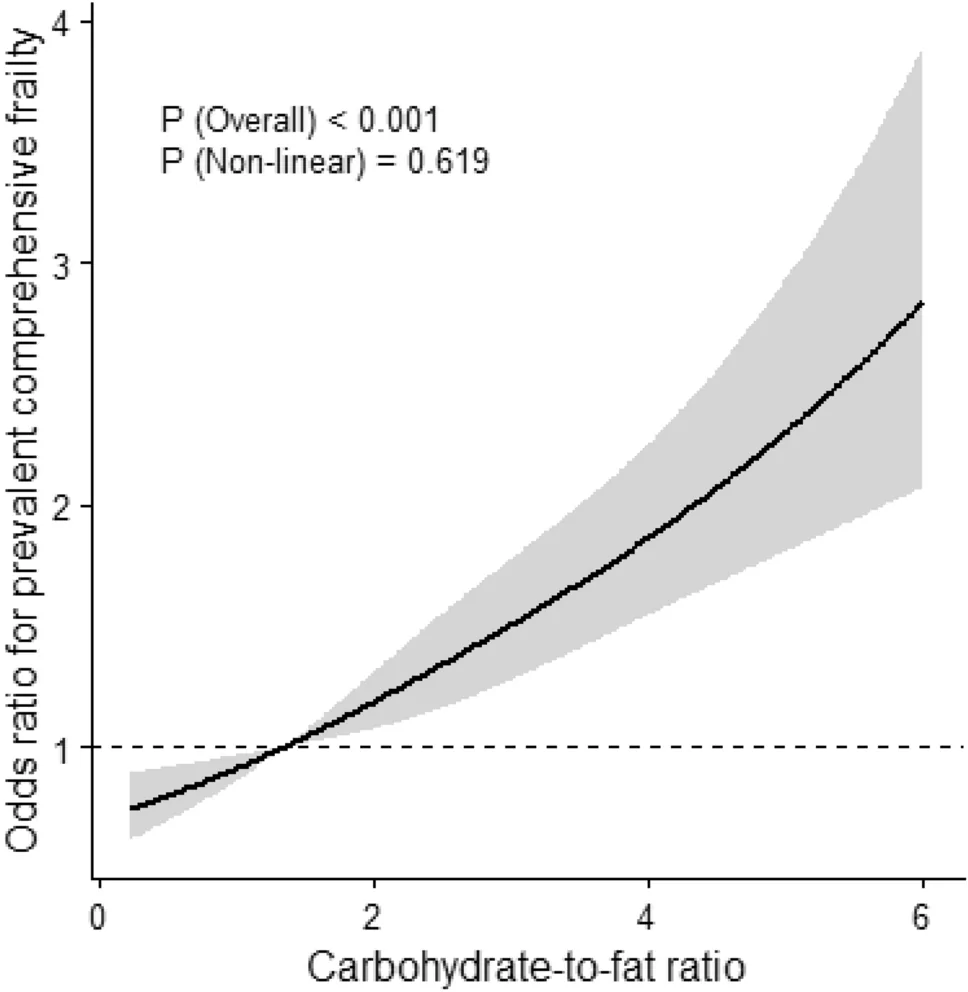
Restricted cubic spline association between the carbohydrate-to-fat energy ratio and prevalent comprehensive frailty. Odds ratios (ORs) and 95% confidence intervals (CIs) were estimated using multivariable logistic regression with restricted cubic splines for the carbohydrate-to-fat energy ratio (CFr). Three knots were placed at the 10th, 50th, and 90th percentiles of the CFr distribution in the overall analytic sample. Comprehensive frailty was defined as a Kihon Checklist score ≥7. The model was adjusted for sex, age, body mass index, total energy intake, alcohol consumption status, smoking status, history of disease, family structure, educational attainment, economic difficulty, population density, and self-rated health. The reference value was set at CFr = 1.34, corresponding to the median CFr within the lowest quartile in the overall analytic sample. The shaded area indicates the 95% CI. *P* for overall association and *P* for nonlinearity were derived from Wald χ^2^ tests for all CFr spline terms and the nonlinear spline component, respectively

Restricted cubic spline models showed a significant positive association between CFr and the prevalence of comprehensive frailty (*P* for overall association <0.001), with no evidence of nonlinearity (*P* for nonlinearity = 0.619) (Fig. 2). Using the median CFr value in the lowest quartile as the reference, the odds of prevalent comprehensive frailty increased approximately linearly with increasing CFr. Similar dose–response patterns were observed when participants were stratified using a cutoff of 1.0 g/kg body weight/day for body-weight-normalized protein intake (*P* for overall association = 0.002 for <1.0 g/kg/day and <0.001 for ≥1.0 g/kg/day) (Supplementary Figure S3).

**Fig. 3:**
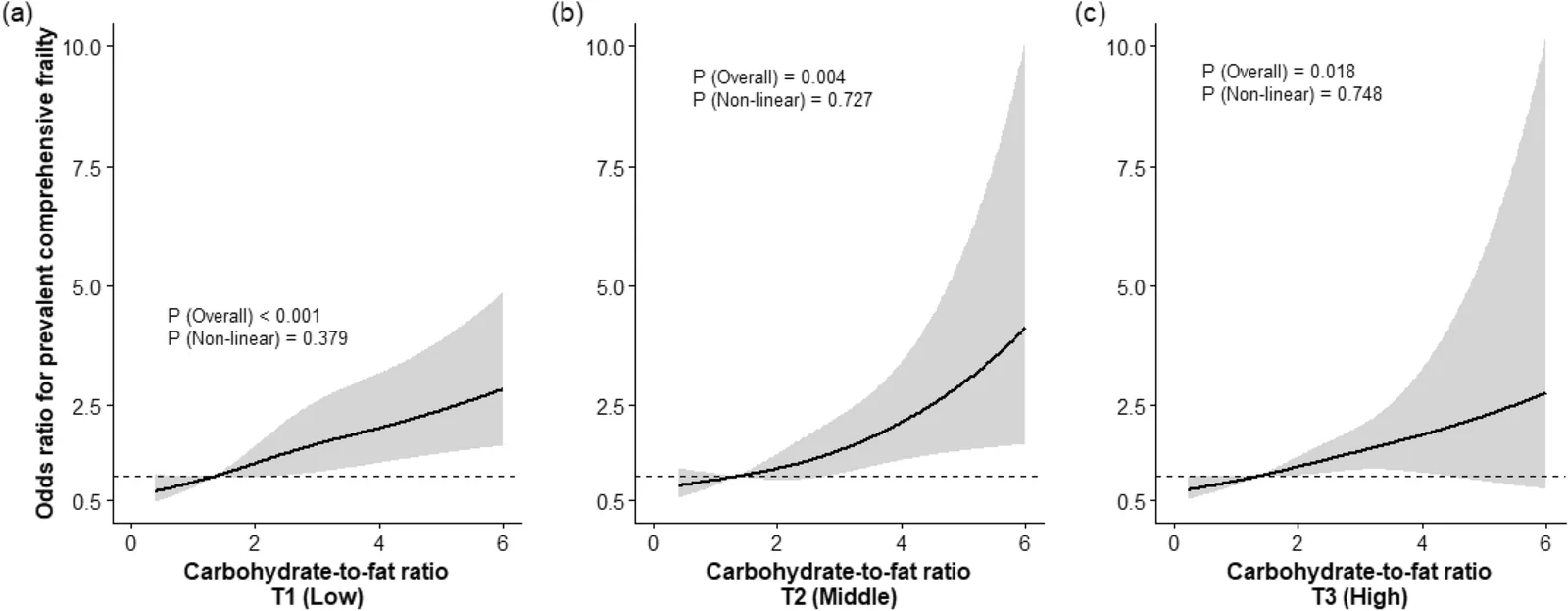
Restricted cubic spline associations between the carbohydrate-to-fat energy ratio and prevalent comprehensive frailty according to sex-specific tertiles of protein intake. Panels A, B, and C represent the lowest (T1), middle (T2), and highest (T3) sex-specific tertiles of protein intake, respectively. ORs and 95% CIs were estimated using multivariable logistic regression with restricted cubic splines for CFr. Three knots were placed at the 10th, 50th, and 90th percentiles of the CFr distribution in the overall analytic sample, and the same knot locations were applied to all protein-intake strata. Comprehensive frailty was defined as a Kihon Checklist score ≥7. Models were adjusted for sex, age, body mass index, total energy intake, alcohol consumption status, smoking status, history of disease, family structure, educational attainment, economic difficulty, population density, and self-rated health. The reference value was set at CFr = 1.34, corresponding to the median CFr within the lowest quartile in the overall analytic sample, and the same reference value was applied to all strata. Shaded areas indicate 95% CIs. *P* for overall association and *P* for nonlinearity were derived from Wald χ^2^ tests for all CFr spline terms and the nonlinear spline component, respectively. Effect modification by protein-intake tertile was evaluated using a likelihood-ratio test comparing models with and without interaction terms between protein-intake tertile and the CFr spline terms (*P* for interaction = 0.93)

Positive dose–response associations were also observed across all sex-specific tertiles of protein intake (*P* for overall association <0.001, 0.004, and 0.018 for the low, middle, and high tertiles, respectively), with no evidence of nonlinearity in any stratum (*P* for nonlinearity = 0.379, 0.727, and 0.748, respectively) (Fig. 3). Formal interaction testing provided no evidence that the association between CFr and comprehensive frailty differed by protein-intake tertile (*P* for interaction = 0.93). Additional sensitivity analyses supported the robustness of the main findings. To minimize the potential influence of alcohol-derived energy on macronutrient energy composition, we repeated the analysis after restricting the study population to non-current drinkers. The positive association between CFr and comprehensive frailty remained evident (P for overall association <0.001), with no evidence of nonlinearity (P for nonlinearity = 0.817) (Supplementary Figure S4).

**Fig. 4:**
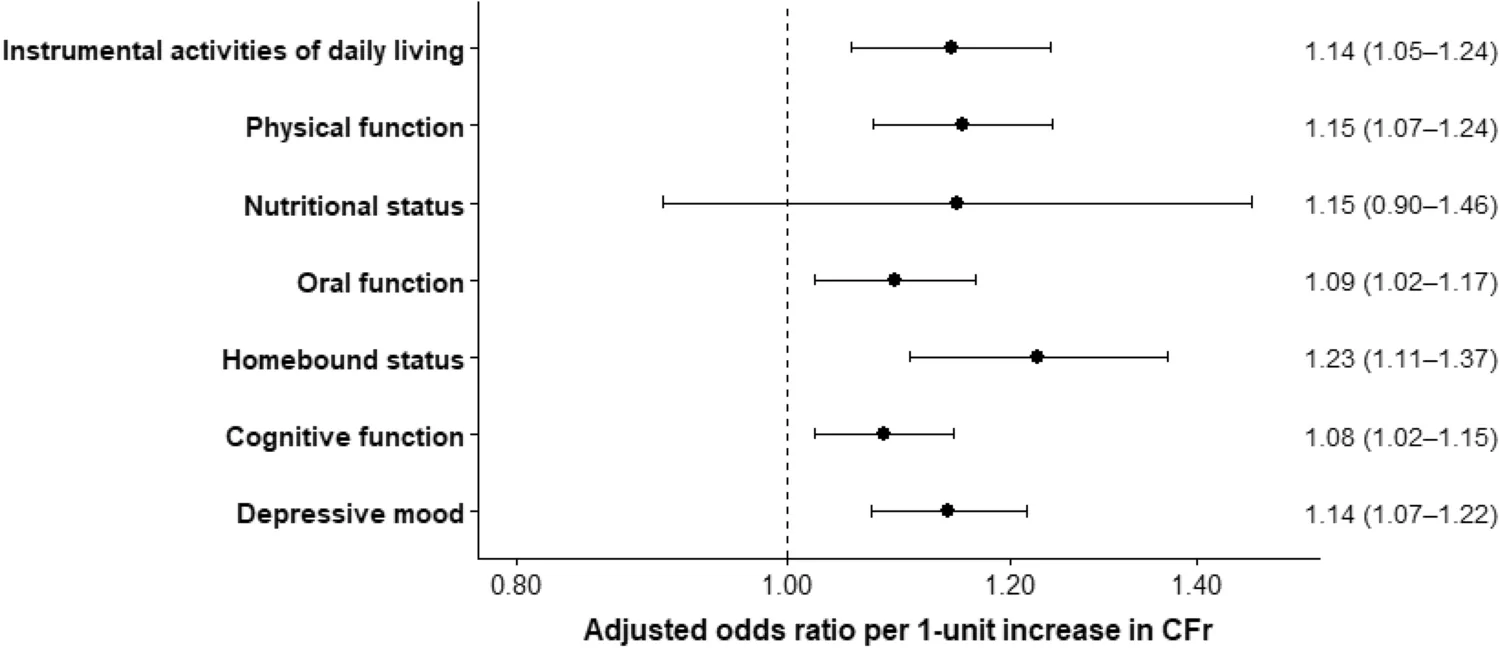
Associations between the carbohydrate-to-fat energy ratio and domain-specific impairments assessed using the Kihon Checklist. Multivariable-adjusted odds ratios (ORs) and 95% confidence intervals (CIs) are shown for impairment in each Kihon Checklist (KCL) domain per 1-unit increase in the carbohydrate-to-fat energy ratio (CFr). Models were adjusted for sex, age, body mass index, total energy intake, alcohol consumption status, smoking status, history of disease, family structure, educational attainment, economic difficulty, population density, and self-rated health. The vertical dashed line indicates an OR of 1.0, and point estimates with 95% CIs are shown numerically on the right. Domain-specific impairments were defined using established KCL cutoffs: instrumental activities of daily living (IADL; Q1–5 ≥ 3), physical function (Q6–10 ≥ 3), nutritional status (Q11–12 = 2), oral function (Q13–15 ≥ 2), housebound/socialization status (Q16: “No”), cognitive function (Q18–20 ≥ 1), and depressive mood (Q21–25 ≥ 2)

In the domain-specific analyses, each 1-unit increase in CFr was associated with higher odds of impairment in instrumental activities of daily living (OR, 1.14; 95% CI, 1.05–1.24), physical function (OR, 1.15; 95% CI, 1.07–1.24), oral function (OR, 1.09; 95% CI, 1.02–1.17), housebound/socialization status (OR, 1.23; 95% CI, 1.11–1.37), cognitive function (OR, 1.08; 95% CI, 1.02–1.15), and depressive mood (OR, 1.14; 95% CI, 1.07–1.22), whereas the association between CFr and nutritional-status impairment was not statistically significant (OR, 1.15; 95% CI, 0.90–1.46) (Fig. 4).

## Discussion

In this cross-sectional study of Japanese community-dwelling older adults, the CFr was positively associated with the prevalence of comprehensive frailty. Across CFr quartiles, multivariable-adjusted ORs of frailty increased monotonically; the highest quartile had approximately 2.0-fold higher odds than the lowest. Consistently, restricted cubic spline models demonstrated a near-linear positive association across the observed CFr range. This pattern was also evident within each tertile of protein intake, indicating that carbohydrate–fat allocation may be relevant even among individuals with high protein intake. Together, these results suggest that data regarding the relative balance between intake of carbohydrates and fats may provide additional information relevant to comprehensive frailty beyond protein intake alone.

The domain-specific analyses showed that the association with higher CFr extended across several components of the KCL and was not limited to nutritional-status or oral-function impairment. Moreover, in an exploratory sensitivity analysis using a modified 20-item KCL outcome that omitted the nutritional-status and oral-function items, the overall association with CFr remained statistically significant. These findings suggest that the observed association was not driven solely by KCL components that conceptually overlap with dietary intake. However, because the modified outcome and its exploratory cutoff have not been formally validated, these results should be interpreted cautiously.

These findings align with a growing body of evidence suggesting that carbohydrate-dominant macronutrient patterns may be unfavorable for frailty. The Korean Frailty and Aging Cohort Study reported that consuming more than 65% of total energy from carbohydrates was associated with a higher prevalence of frailty [18], and higher carbohydrate intake was associated with a worse frailty index in the Baltimore Longitudinal Study of Aging [17]. In addition, a higher dietary carbohydrate-to-fat ratio has been associated with poorer global cognitive performance in older adults at risk of dementia [19], indicating that the relative allocation of energy between carbohydrate and fat may also be relevant to health domains beyond physical frailty. Taken together, these findings support further investigation of carbohydrate–fat energy allocation in relation to comprehensive frailty, including among older adults with relatively high protein intake. Prospective and interventional studies that jointly consider protein, fat, and carbohydrate intake are needed to clarify the temporal relationship and the role of overall macronutrient balance in frailty.

Across protein-intake tertiles, higher CFr was associated with higher odds of prevalent comprehensive frailty in both quartile-based logistic regression and restricted cubic spline analyses, with approximately linear associations and no evidence of effect modification by protein-intake level. This pattern suggests that the observed association was not confined to a particular protein-intake stratum. CFr was also generally lower in the highest protein-intake tertile. Given that previous studies have emphasized the importance of adequate protein intake in relation to frailty [8, 32], these findings suggest that the relative distribution of energy between carbohydrates and fats may provide additional information when characterizing dietary patterns associated with frailty.

Reverse causation should also be considered. Frail older adults may experience reduced appetite, oral dysfunction, difficulty shopping for or preparing food, financial constraints, or comorbidities that influence food choices and macronutrient intake, potentially resulting in a higher CFr. In addition, the KCL includes nutritional-status and oral-function items that may conceptually overlap with dietary intake. Although the positive association between CFr and frailty persisted in an exploratory sensitivity analysis using a modified KCL that excluded the nutritional-status and oral-function items (questions 11–15) (Supplementary Figure S5), this finding did not establish temporality. The modified outcome and its exploratory cutoff have not been formally validated; thus, the analysis should be interpreted cautiously and does not exclude reverse causation.

Accordingly, these findings should be regarded as hypothesis-generating and do not establish causality. CFr may reflect higher carbohydrate intake, lower fat intake, or both, but dose not provide information on the quality or food sources of either macronutrient [33–35]. Therefore, these results do not support recommendations for older adults to lower their CFr, restrict carbohydrate intake, or increase fat intake, and current clinical or public health recommendations should not be changed solely on the basis of this study. Rather, the findings suggest that overall macronutrient balance, in addition to adequate protein intake, may warrant consideration in future research on frailty prevention. Prospective and interventional studies are needed to examine macronutrient balance together with diet quality, including carbohydrate and fat quality and food sources.

This study has several limitations. First, its cross-sectional design precludes causal inference and raises the possibility of reverse causation, such as prodromal frailty altering appetite or food choices and thereby shifting macronutrient balance. Second, dietary exposures were self-reported and assessed at a single time point; thus, measurement error and within-person variation may have attenuated or distorted the observed associations. Third, although the models were adjusted for a comprehensive set of covariates, residual and unmeasured confounding cannot be excluded. Fourth, CFr is an informative summary of relative energy allocation; however, as a ratio, it cannot determine whether the observed associations reflect a higher contribution of carbohydrates to energy intake, a lower contribution of fat, or both, nor does it identify the specific isocaloric substitution involved. Formal compositional data analysis using Aitchison geometry or isocaloric substitution models may help refine interpretation in future studies. Fifth, the exclusion of participants with missing or invalid anthropometric data or incomplete KCL responses may have introduced selection bias if the excluded participants differed systematically from those included in the analytic sample. Finally, the participants were Japanese community-dwelling older adults, and the findings may not be generalizable to populations with different dietary patterns, food environments, or ethnic backgrounds.

## Conclusion

In this cross-sectional sample of 5679 older adults, a higher CFr was associated with a greater prevalence of comprehensive frailty, with broadly similar associations across protein-intake levels. Prospective and interventional studies are needed to clarify the temporal relationship and to examine how carbohydrate and fat quantity, quality, and food sources relate to frailty across different levels of protein intake.

## Supplementary Information

Below is the link to the electronic supplementary material.

Supplementary file1 (PPTX 594 kb)

Supplementary file2 (DOCX 21 kb)

## Data Availability

The datasets analyzed during the current study are not publicly available because they contain potentially identifiable participant information. Data may be available from the Kyoto-Kameoka Study Group on reasonable request to the corresponding data manager, Yosuke Yamada (yosuke.yamada.c1@tohoku.ac.jp), subject to approval by the relevant ethics committees and institutional data-sharing policies.

## References

[1] Rockwood K (2005) Frailty and its definition: a worthy challenge. J Am Geriatr Soc 53(6):1069–1070. 10.1111/j.1532-5415.2005.53312.x 15935037

[2] Clegg A, Young J, Iliffe S et al (2013) Frailty in elderly people. Lancet 381(9868):752–762. 10.1016/S0140-6736(12)62167-9 PMC4098658 23395245

[3] Lorenzo-Lopez L, Maseda A, de Labra C et al (2017) Nutritional determinants of frailty in older adults: a systematic review. BMC Geriatr 17(1):108. 10.1186/s12877-017-0496-2 PMC5433026 28506216

[4] Tagawa R, Watanabe D, Ito K et al (2020) Dose-response relationship between protein intake and muscle mass increase: a systematic review and meta-analysis of randomized controlled trials. Nutr Rev. 10.1093/nutrit/nuaa104 PMC7727026 33300582

[5] Coelho-Junior HJ, Marzetti E, Picca A et al (2020) Protein intake and frailty: a matter of quantity, quality, and timing. Nutrients. 10.3390/nu12102915 PMC7598653 32977714

[6] Kobayashi S, Suga H, Sasaki S et al (2017) Diet with a combination of high protein and high total antioxidant capacity is strongly associated with low prevalence of frailty among old Japanese women: a multicenter cross-sectional study. Nutr J 16(1):29. 10.1186/s12937-017-0250-9 PMC5429552 28499379

[7] Nanri H, Yamada Y, Yoshida T et al (2018) Sex difference in the association between protein intake and frailty: assessed using the Kihon checklist indexes among older adults. J Am Med Dir Assoc 19(9):801–805. 10.1016/j.jamda.2018.04.005 29861193

[8] Nanri H, Watanabe D, Yoshida T et al (2022) Adequate protein intake on comprehensive frailty in older adults: Kyoto-Kameoka study. J Nutr Health Aging 26(2):161–168. 10.1007/s12603-022-1740-9 PMC12878383 35166309

[9] Lieberman HR, Fulgoni VL, Agarwal S et al (2020) Protein intake is more stable than carbohydrate or fat intake across various US demographic groups and international populations. Am J Clin Nutr 112(1):180–186. 10.1093/ajcn/nqaa044 PMC7326590 32297956

[10] Bilsborough S, Mann N (2006) A review of issues of dietary protein intake in humans. Int J Sport Nutr Exerc Metab 16(2):129–152. 10.1123/ijsnem.16.2.129 16779921

[11] Ashtary-Larky D, Bagheri R, Bavi H et al (2022) Ketogenic diets, physical activity and body composition: a review. Br J Nutr 127(12):1898–1920. 10.1017/S0007114521002609 PMC9244428 34250885

[12] Dehghan M, Mente A, Zhang X et al (2017) Associations of fats and carbohydrate intake with cardiovascular disease and mortality in 18 countries from five continents (PURE): a prospective cohort study. Lancet 390(10107):2050–2062. 10.1016/S0140-6736(17)32252-3 28864332

[13] U.S. Department of Agriculture ARS. What We Eat in America (WWEIA), NHANES 2017–2018: Table 5. Percentages of Energy from Protein, Carbohydrate, Fat, and Alcohol Washington, DCn.d. Accessed on 16 Sept 2025

[14] Ministry of Health, Labour and Welfare. Report of the National Health and Nutrition Survey, Japan, 2023. Tokyo, Japan, 2025.

[15] Sandoval-Insausti H, Perez-Tasigchana RF, Lopez-Garcia E et al (2016) Macronutrients intake and incident frailty in older adults: a prospective cohort study. J Gerontol A Biol Sci Med Sci 71(10):1329–1334. 10.1093/gerona/glw033 26946103

[16] Verspoor E, Voortman T, van Rooij FJA et al (2020) Macronutrient intake and frailty: the Rotterdam study. Eur J Nutr 59(7):2919–2928. 10.1007/s00394-019-02131-0 PMC7501120 31728680

[17] Tanaka T, Kafyra M, Jin Y et al (2022) Quality specific associations of carbohydrate consumption and frailty index. Nutrients. 10.3390/nu14235072 PMC9736578 36501101

[18] Yang N, Lee Y, Kim MK et al (2023) Macronutrients intake and physical frailty in Korean older adults: a cohort-based cross-sectional study. Geriatr Gerontol Int 23(7):478–485. 10.1111/ggi.14597 37246816

[19] Norgren J, Sindi S, Sandebring-Matton A et al (2023) The dietary carbohydrate/fat-ratio and cognitive performance: panel analyses in older adults at risk for dementia. Curr Dev Nutr 7(6):100096. 10.1016/j.cdnut.2023.100096 PMC10236460 37275847

[20] Yamada Y, Nanri H, Watanabe Y et al (2017) Prevalence of frailty assessed by Fried and Kihon checklist indexes in a prospective cohort study: design and demographics of the Kyoto-Kameoka longitudinal study. J Am Med Dir Assoc 18(8):733 e7-833. 10.1016/j.jamda.2017.02.022 28501417

[21] Goto C, Tokudome Y, Imaeda N, Takekuma K, Kuriki K, Igarashi F, Ikeda M, Tokudome S (2006) Validation study of fatty acid consumption assessed with a short food frequency questionnaire against plasma concentration in middle-aged Japanese people. Scand J Food Nutr 50(2):77–82

[22] Imaeda N, Goto C, Tokudome Y et al (2007) Reproducibility of a short food frequency questionnaire for Japanese general population. J Epidemiol 17(3):100–107 10.2188/jea.17.100 PMC7058456 17545697

[23] Tokudome S, Goto C, Imaeda N et al (2004) Development of a data-based short food frequency questionnaire for assessing nutrient intake by middle-aged Japanese. Asian Pac J Cancer Prev 5(1):40–43 15075003

[24] Tokudome Y, Goto C, Imaeda N et al (2005) Relative validity of a short food frequency questionnaire for assessing nutrient intake versus three-day weighed diet records in middle-aged Japanese. J Epidemiol 15(4):135–145 10.2188/jea.15.135 PMC7851066 16141632

[25] Watanabe D, Nanri H, Yoshida T et al (2019) Validation of energy and nutrition intake in Japanese elderly individuals estimated based on a short food frequency questionnaire compared against a 7-day dietary record: the Kyoto-Kameoka study. Nutrients 11(3):688 10.3390/nu11030688 PMC6471352 30909514

[26] Science and Technology Agency of Japan. Standard Tables of Food Composition in Japan, 5th rev. ed.; JPB, Ministry of Finance: Tokyo, Japan, 2001.

[27] Zhu W, Chen Q, Li D et al (2026) Low-carbohydrate, high-fat diet is inversely associated with the triglyceride-glucose index: evidence from NHANES 1999–2018. Eur J Med Res. 10.1186/s40001-026-04126-0 41782055

[28] Arai H, Satake S (2015) English translation of the Kihon Checklist. Geriatr Gerontol Int 15(4):518–519. 10.1111/ggi.12397 25828791

[29] Maseda A, Lorenzo-Lopez L, Lopez-Lopez R et al (2017) Spanish translation of the Kihon Checklist (frailty index). Geriatr Gerontol Int 17(3):515–517. 10.1111/ggi.12892 28345232

[30] Satake S, Senda K, Hong YJ et al (2016) Validity of the Kihon Checklist for assessing frailty status. Geriatr Gerontol Int 16(6):709–715. 10.1111/ggi.12543 26171645

[31] Watanabe D, Yoshida T, Watanabe Y et al (2022) Validation of the Kihon Checklist and the frailty screening index for frailty defined by the phenotype model in older Japanese adults. BMC Geriatr 22(1):478. 10.1186/s12877-022-03177-2 PMC9164897 35658843

[32] Coelho-Júnior HJ, Rodrigues B, Uchida M et al (2018) Low protein intake is associated with frailty in older adults: a systematic review and meta-analysis of observational studies. Nutrients. 10.3390/nu10091334 PMC6165078 30235893

[33] Sacks FM, Lichtenstein AH, Wu JHY et al (2017) Dietary fats and cardiovascular disease: a presidential advisory from the American Heart Association. Circulation 136(3):e1–e23. 10.1161/CIR.0000000000000510 28620111

[34] Te Morenga L, Mallard S, Mann J (2013 ) Dietary sugars and body weight: systematic review and meta-analyses of randomised controlled trials and cohort studies. BMJ 346:e7492. 10.1136/bmj.e7492 23321486

[35] Reynolds A, Mann J, Cummings J et al (2019) Carbohydrate quality and human health: a series of systematic reviews and meta-analyses. Lancet 393(10170):434–445. 10.1016/S0140-6736(18)31809-9 30638909

