# Supplementary material for: Carbohydrate-to-fat ratio is positively associated with comprehensive frailty across protein-intake levels in older adults
Source: Eur J Nutr. 2026 Oct 7;65(8):271. doi: 10.1007/s00394-026-04099-0 (PMC13646010; doi:10.1007/s00394-026-04099-0)
Supplement: Supplementary file 1 [file 394_2026_4099_MOESM2_ESM.docx]

Supplementary Table S1. Distribution of total Kihon Checklist scores overall and according to carbohydrate-to-fat ratio quartile and sex-specific protein-intake tertile.

| Group | Category | n | Kihon Checklist score | | | KCL ≥7, n (%) |
| --- | --- | --- | --- | --- | --- | --- |
|  |  |  | Mean (SD) | Median (IQR) | Range |  |
| Overall | All participants | 5,679 | 5.05 (4.19) | 4 (2-7) | 0-23 | 1,705 (30.0) |
| Carbohydrate-to-fat ratio quartile | Q1 | 1,420 | 4.62 (4.06) | 4 (1-7) | 0-23 | 370 (26.1) |
|  | Q2 | 1,420 | 4.87 (4.09) | 4 (2-7) | 0-23 | 380 (26.8) |
|  | Q3 | 1,419 | 5.11 (4.19) | 4 (2-7) | 0-22 | 443 (31.2) |
|  | Q4 | 1,420 | 5.59 (4.36) | 5 (2-8) | 0-23 | 512 (36.1) |
| Protein intake tertile | T1 | 1,894 | 5.31 (4.24) | 4 (2-8) | 0-23 | 625 (33.0) |
|  | T2 | 1,892 | 4.80 (4.09) | 4 (2-7) | 0-23 | 524 (27.7) |
|  | T3 | 1,893 | 5.02 (4.23) | 4 (2-7) | 0-23 | 556 (29.4) |

Values are presented as mean (SD), median (IQR), range, or n (%), as indicated. IQR, interquartile range; KCL, Kihon Checklist; SD, standard deviation. Carbohydrate-to-fat ratio quartiles were defined using cut-points corresponding to the 25th, 50th, and 75th percentiles of the overall analytic sample (1.69, 2.26, and 3.02, respectively). Protein-intake tertiles were defined separately for men and women using sex-specific cut-points (% energy): 10.9% and 12.4% for men and 12.1% and 14.0% for women.
